# Pacybara: Accurate long-read sequencing for barcoded mutagenized allelic libraries

**DOI:** 10.1101/2023.02.22.529427

**Published:** 2023-02-23

**Authors:** Jochen Weile, Atina G. Cote, Nishka Kishore, Daniel Tabet, Warren van Loggerenberg, Ashyad Rayhan, Frederick P Roth

**Affiliations:** 1 Lunenfeld-Tanenbaum Research Institute, Sinai Health, Toronto, ON, M5G 1X5, Canada; 2 The Donnelly Centre, University of Toronto, Toronto, ON, M5S 3E1, Canada; 3 Department of Molecular Genetics, University of Toronto, Toronto, ON, M5S 3E1, Canada; 4 Department of Computer Science, University of Toronto, Toronto, ON, M5S 2E4

## Abstract

Long read sequencing technologies, an attractive solution for many applications, usually suffer from higher error rates. Alignment of multiple reads can improve base-calling accuracy, but some applications, e.g. the sequencing of mutagenized libraries where multiple distinct clones differ by one or few variants, require the use of barcodes or unique molecular identifiers. Unfortunately, not only can sequencing errors interfere with correct barcode identification, but a given barcode sequence may be linked to multiple independent clones within a given library.

Here we focus on the target application of sequencing mutagenized libraries in the context of multiplexed assays of variant effects (MAVEs). MAVEs are increasingly used to create comprehensive genotype-phenotype maps that can aid clinical variant interpretation. Many MAVE methods use barcoded mutant libraries and thus require the accurate association of barcode with genotype, e.g. using long-read sequencing. Existing pipelines do not account for inaccurate sequencing or non-unique barcodes. Here, we describe Pacybara, which handles these issues by clustering long reads based on the similarities of (error-prone) barcodes while detecting the association of a single barcode with multiple genotypes. Pacybara also detects recombinant (chimeric) clones and reduces false positive indel calls. In an example application, we show that Pacybara increases the sensitivity of a MAVE-derived missense variant effect map.

## Introduction

Multiplexed Assays of Variant Effect (MAVEs) often involve the use of clone libraries in which each sequenced allele is associated with a barcode (Tabet et al. 2022), requiring determination of full-length amplicon sequences. Long-read sequencing technologies such as Pacbio Sequel II and Oxford Nanopore have become an attractive alternative to short-read assembly methods (Hiatt et al. 2010; Weile et al. 2017) .

The first pipeline for long-read barcode library assembly was AssemblyByPacbio (ABP) (Matreyek et al. 2018). ABP extracts barcode sequences from each read, groups reads by identical barcodes, and uses the highest quality read in each group as the genotype. Recently, the alternative pipeline PacRat was published (Yeh et al. 2022), which calculates a multiple sequence alignment-based consensus for each read group sharing a barcodes.

At least three problems have remained unsolved: First, sequencing errors in the barcode can cause a single clone to appear as two or more distinct clones. Although an error-aware clustering method for short barcode sequences (Zhao et al. 2018) addressed a similar issue for short read barcode identification (BarSeq), it does not consider clonality of genotypes beyond the barcode itself. Second, a barcode sequence may be “non-unique”, in the sense that the same sequence has been associated with multiple different-genotype clones within a given library. Third, problems arising during library preparation, e.g. PCR crossover events, can yield consensus genotypes that are recombinant chimeras of wild-type or other variant genotypes. Karst and colleagues have described a double-barcode method to detect chimeras, but this approach was not optimal where clones might differ by only a few SNVs, as is the case for mutagenized libraries used in MAVEs (Karst et al. 2021).

To address these issues, we expanded upon the above-cited clustering approaches using not only the barcode sequences from each read but also the full set of candidate variants and their quality metrics. Although initial stages of the clustering process, like previous methods, focused on merging pairs of reads with identical candidate barcodes, Pacybara accounts for variant quality and the extent of overlap between candidate variants. Pacybara then further considers merging of reads with similar but non-identical barcodes.

## Methods

Pacybara is designed to run on high-performance computing clusters. As such it consists of a main executable that deploys and supervises individual jobs on the cluster nodes and then collates the results. In the first step of processing, Pacbio reads are distributed across jobs where they are aligned to the reference sequence. From the alignments, barcode sequences and lists of candidate variants (i.e. basecalls that differ from the reference sequence and could either be variants or sequencing errors) are extracted, including their respective quality scores. For libraries designed for multiple barcodes per molecule, the latter are combined to form a single “virtual barcode” each.

Distant reads that have no chance of ultimately being clustered together can then be sent for processing in parallel in different jobs nodes. Therefore, all extracted barcodes are aligned against each other to ‘connect’ reads within a threshold edit distance. Each job can then process the reads from one or more of the connected components in the resulting graph. A given job seeks to cluster similar reads originating from the same clonal DNA (on the basis of carrying the same barcode and having a set of variants that differs no more than what might be expected from sequencing errors).

Clustering begins by considering sets of reads with identical barcode sequences. Within each identical-barcode set, putative sequencing errors are excluded by filtering out candidate variants seen in only one read with a below-threshold quality score. Then for each identical-barcode read set, a graph is constructed such that each node corresponds to a read, and an edge is added between two reads if either both are WT or if they harbour a sufficient similar set of candidate variants (defined by a Jaccard index threshold, default 0.2). A “seed cluster” is then formed for each graph component (including nodes without edges). Cluster definitions, initially defined by the set of seed clusters, are updated in a cluster-merging process. Pairs of clusters, chosen by the minimum edit distance of their respective member reads, are iteratively considered as candidate clusters. After filtering sequencing errors from this candidate cluster as above, the candidate cluster is accepted if all remaining reads in both clusters are WT, or if both of the following are true: a) sets of remaining candidate variants in the respective pair of clusters have an above-threshold Jaccard coefficient (default 0.2), and that b) the pair of clusters has sufficient-divergent sizes, as might be expected if one cluster represents a subset of reads derived from the same clone, but with more base-calling errors in the barcode. Here the clusters were considered to have sufficiently-divergent sizes if |log2(size_1_/size_2_)| > editDistance, similar to an approach described previously for short-read barcode clustering (Zhao et al. 2018).

For each cluster in the final set, consensus barcodes are derived, variants are filtered as above to remove putative base-calling errors, and (for coding sequences) remaining variants are translated to protein consequences.

## Results and Discussion

We evaluated performance of both Pacybara and the previously-established method PacRat, which forms consensus sequences for each set of reads with 100% barcode sequence identity, without considering potential sequencing errors. Both tools were evaluated on a barcoded mutagenized open reading frame library, designed for use in a MAVE of the human LDLR protein.

**Figure 1:**
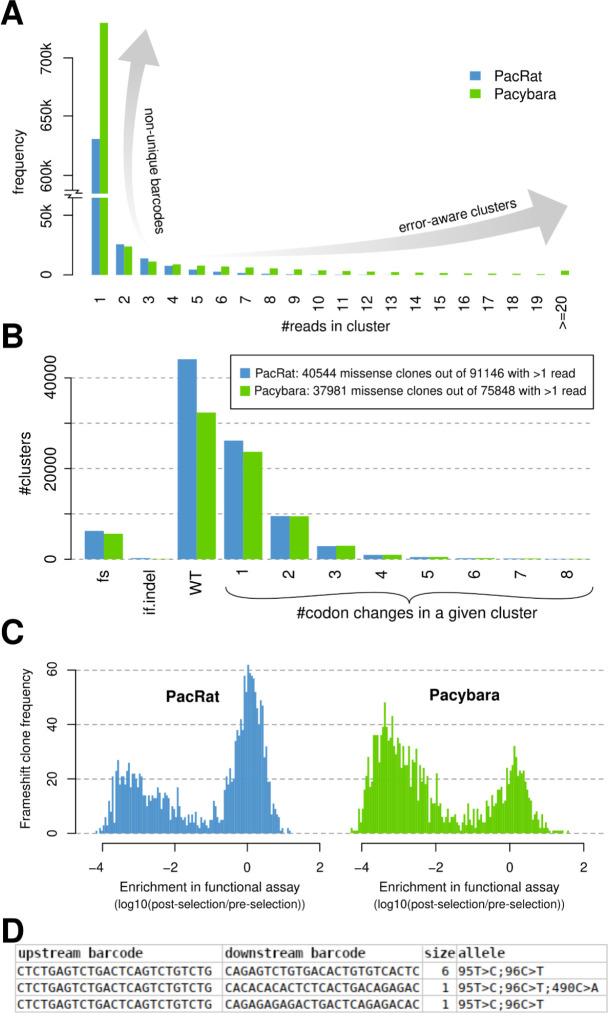
A: Comparison of cluster size frequencies. Both PacRat and Pacybara were applied to the same dataset (a mutagenized library of LDLR flanked by two barcodes). B: Comparison of the frequencies of clusters carrying different types of alleles. fs = frameshift; if.indel = in-frame insertion or deletion; WT = wildtype allele; 1–8 = carrying the corresponding number of codon changes. C: Histogram of functionality scores assigned to clones (i.e. clusters) identified by PacRat and Pacybara respectively. Functionality scores represent enrichment (log-ratio of post-selection to pre-selection counts) in a fluorescence-based LDLR uptake assay and act as a proxy for protein function. Scores near 0 indicate normal protein function, while negative scores indicate protein function disruption. D: Three clusters with identical upstream barcodes and alleles but different downstream barcodes occurring in the mutagenized LDLR library, indicating likely PCR chimeras. “Size” refers to the number of reads in the cluster.

Differences between Pacybara and PacRat are immediately apparent in the distribution of cluster sizes (Figure 1A). Pacybara yields a relatively larger number of singleton clusters, which we attribute to fewer erroneous merges between clones with non-unique barcodes by considering the overlap between candidate variants before merging. Pacybara also exhibits a longer tail of high-read-count clusters, likely because Pacybara can merge reads with barcodes that differ only due to sequencing error.

Pacybara also yielded relatively fewer apparent wildtype clusters, presumably because it rejected merges between clusters with non-unique barcodes that might otherwise dilute the impact of candidate variants and produce wild-type consensus sequences. Pacybara registered missense variants in 50.1% of non-singleton clusters as opposed to 44.5% for PacRat (Figure 1B).

To validate whether the barcode-genotype associations produced by Pacybara are indeed more accurate, we tested them in terms of their impact on protein function. As frameshift variants are likely to damage protein function, we evaluated the functionality scores of apparently-frameshift-containing clones that were derived either from PacRat or Pacybara. Functional scores were calculated based on depletion in a fluorescence-based LDLR-uptake assay. Frameshift-containing clones defined by Pacybara were substantially more likely to be deleterious than those defined by PacRat. (Figure 1C)

The inclusion of virtual barcodes that concatenate barcodes from both flanks of the same construct allows Pacybara to also identify putative PCR crossover events. Indeed, we identified multiple sets of clusters in which upstream barcodes were identical and a partial overlap in genotype existed, but which had entirely different downstream barcodes, confirming the value of dual flanking barcodes. (Figure 1D)

In summary, we developed the application Pacybara to process long-read data from a library of barcoded mutagenized clones, and found that it successfully detects non-unique barcodes and PCR crossover events, combines reads with barcodes that differ only due to sequencing error, reduces false-positive indel calls, and improves the quality of a downstream MAVE experiment.

## Supplementary Material

Supplement 1

## Data Availability

Pacybara is freely available at https://github.com/rothlab/pacybara. It is implemented using R, Python and bash for Linux, with both a single-threaded implementation and, for GNU/Linux clusters that use Slurm or PBS schedulers, a multi-node version.
