# Supplementary material for: Pacybara: Accurate long-read sequencing for barcoded mutagenized allelic libraries
Source: bioRxiv. 2023 Feb 23:2023.02.22.529427. Preprint. [Version 1] doi: 10.1101/2023.02.22.529427 (PMC9980134; doi:10.1101/2023.02.22.529427)
Supplement: Supplementary file 1 [file media-1.pdf]

Pacybara: Accurate long-read sequencing for barcoded mutagenized allelic libraries

Supplementary Materials

Pseudocode of the clustering approach:

```
function filterSeqError(qualityMatrix):
  #qualityMatrix has rows for reads and columns for candidate variants
  #where a candidate variant is observed
  #(with total quality score above threshold) it returns the Q-score,
  #otherwise values are undefined
  passedFilter = []
  for i in 1 to numRows(qualityMatrix) :
    numObserved = count(defined(qualityMatrix[i][]))
    if numObserved > 1 or sum(qualityMatrix[i][]) > minQual:
      passedFilter = [passedFilter, i]
  return qualityMatrix[passedFilter][]

# SEED CLUSTERING
#iterate over pairs at edit distance 0
for readSet in readSetsWithIdenticalBarcodes:
  qualMat = qualityMatrix(readSet)
  qualMat = filterSeqError(qualMat)
  #iterate over pairs of reads in readSet
  for i in 2 to length(readSet) :
      read1 = readSet[i]
      variants1 = which(defined(qualMat[][i]))
      for j in 1 to i-1:
        read2 = readSet[j]
        variants2 = which(defined(qualMat[][j]))
        inters = intersection(variants1,variants2)
        union = union(variants1,variants2)
        #if both are WT or thresholds are passed, accept the merge.
        if union == 0 || (inters/union >= minJaccard && inters >= minMatches):
          mergeClustersOf(read1,read2)

# MAIN CLUSTERING
for editDistance in 1 to maxDist:
  for (read1,read2) in getReadPairsAtBarcodeDistance(editDistance):
      #fetch the associated cluster ids
      cluster1 = getClusterForRead(read1)
      cluster2 = getClusterForRead(read2)
      #if we are already in the same cluster, we can skip
```

```
if (cluster1 == cluster2):
  next
#compare sizes (cf. Zhao et al.)
sizeDisproportion = abs(log2(size(cluster1)/size(cluster2)))
#if the sizes are sufficiently different
#(ad hoc threshold set to require another
#log difference for each increase in edit distance),
#then we have fulfilled the first criterion
if sizeDisproportion >= editDistance:
  #check overlap as before.
  qualMat = qualityMatrix(join(cluster,cluster2))
  qualMat = filterSeqError(qualMat)
  variants1 = which(defined(qualMat[][cluster1]))
  variants2 = which(defined(qualMat[][cluster2]))
  inters = intersection(variants1,variants2)
  union = union(variants1,variants2)
  #if both are WT or thresholds
  #(for Jaccard similarity and absolute number of
  #matching candidate variants) are passed, accept the merge.
  if union == 0 or (inters/union >= minJaccard and inters >= minMatches):
    mergeClusters(cluster1,cluster2)
```
